# Combined endoscopic salvage: pancreatoscopy and endoscopic ultrasound-guided pancreatic duct drainage for synchronous postoperative pancreatic fistulas

**DOI:** 10.1055/a-2854-6654

**Published:** 2026-04-30

**Authors:** Zecan Shi, Zheng Jin, Haojie He, Weigang Gu, Qifeng Lou, Xiaofeng Zhang, Hangbin Jin

**Affiliations:** 1 The Fourth School of Clinical Medicine 74630 Zhejiang Chinese Medical University, Hangzhou First Peopleʼs HospitalHangzhouChina; 2 Department of GastroenterologyAffiliated Hangzhou First Peopleʼs Hospital, School of Medicine, Westlake UniversityHangzhouChina; 3 Key Laboratory of Integrated Traditional Chinese and Western Medicine for Biliary and Pancreatic Diseases of Zhejiang ProvinceHangzhouChina; 4 Hangzhou Institute of Digestive DiseasesHangzhouChina

Postoperative pancreatic fistula is a significant complication of pancreatic surgery and potentially leads to life-threatening consequences 1 . While endoscopic retrograde cholangiopancreatography is the preferred endoscopic treatment option 2 , postoperative anatomical changes often result in challenging strictures of the pancreatic duct (PD), particularly in cases involving multifocal lesions.

**Fig. 1:**
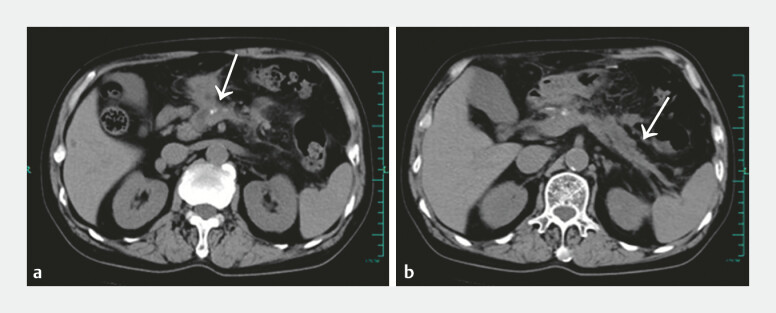
Abdominal enhanced CT images. **a** An exudate in the surgical area of the pancreatic body. **b** Mild dilation of the main PD in the tail of the pancreas. CT, computed tomography; PD, pancreatic duct drainage.

We report a case of dual pancreatic fistulas (body and tail) following resection of an intraductal papillary mucinous neoplasm, refractory to conservative management ( Fig. 1 ).

First, to address the fistula in the pancreatic body, transpapillary drainage was attempted. Cannulation via the major papilla failed because the guidewire could not traverse a severe stricture at the pancreatic neck. Consequently, we proceeded with minor papilla cannulation. Under the guidance of a 7.5-Fr digital single-operator pancreatoscope, the precise location of the disruption was identified, and a 7-Fr plastic stent was placed to bridge the leakage site ( Video 1 ).

Combined endoscopic salvage: Pancreatoscopy and endoscopic ultrasound-guided pancreatic duct drainage for synchronous postoperative pancreatic fistula.Video 1

**Fig. 2:**
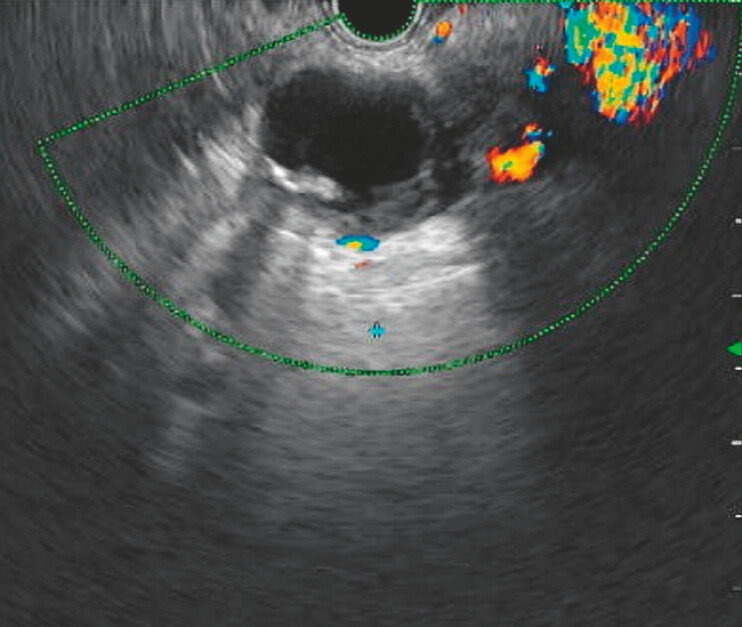
EUS images reveal dilation of the main PD in the tail of the pancreas, along with a fluid-attenuated area in the pancreatic body. EUS, endoscopic ultrasound; PD, pancreatic duct drainage.

**Fig. 3:**
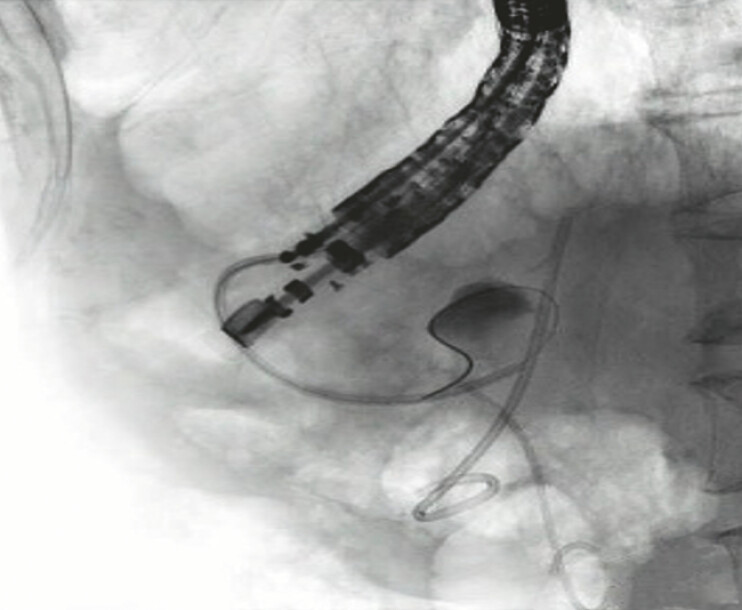
Placement of a nasopancreatic tube into the fistula cavity under fluoroscopic guidance.

Second, the disconnected fistula in the pancreatic tail was managed using endoscopic ultrasound (EUS)-guided pancreatic duct drainage (EUS-PD). The dilated main pancreatic duct was punctured via the stomach, and a naso-pancreatic drain was initially deployed ( Fig. 2 , Fig. 3 ).

**Fig. 4:**
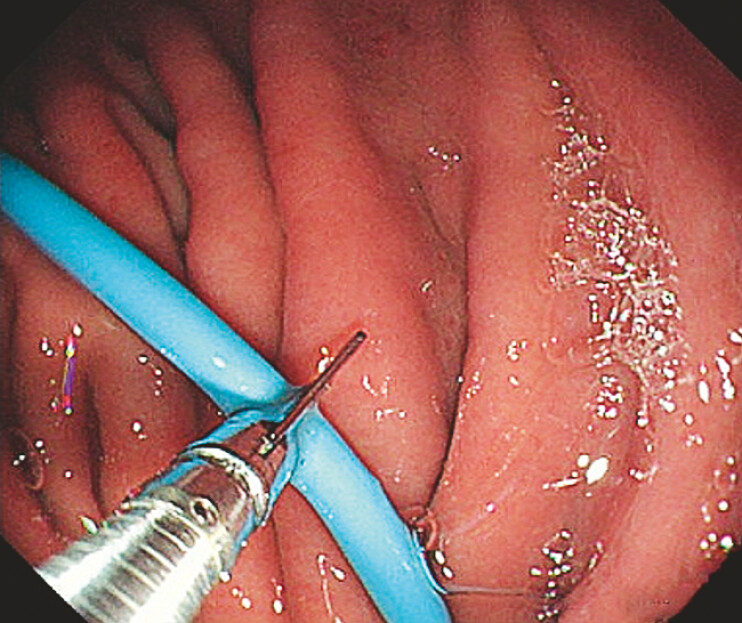
Division of the nasopancreatic tube under direct visualization to create an internal stent.

This drain was subsequently shortened endoscopically to serve as an internal stent ( Fig. 4 ).

**Fig. 5:**
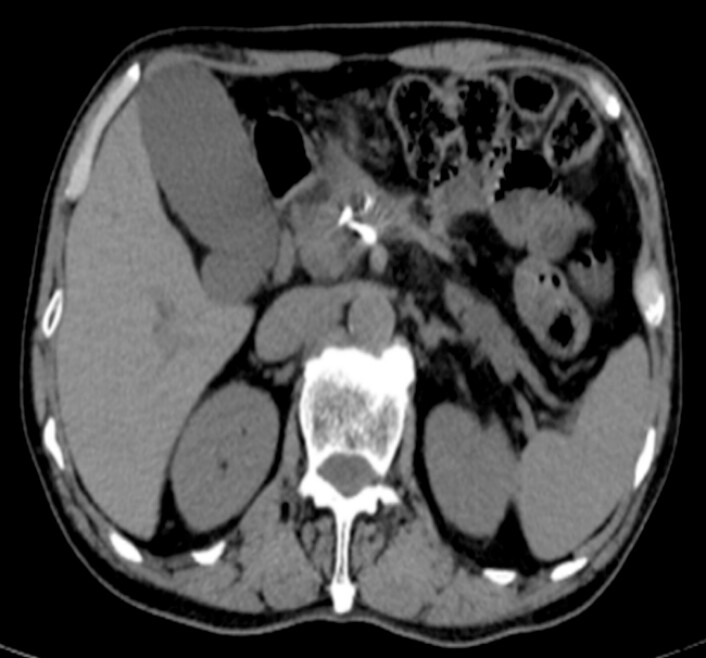
CT imaging demonstrates a significant reduction in the fistula cavity within the body of the pancreas, with no significant dilation observed in the main PD in the tail of the pancreas. CT, computed tomography; PD, pancreatic duct drainage.

Follow-up imaging confirmed the resolution of fluid collections and fistula closure ( Fig. 5 ).

This case demonstrates the efficacy of a combined approach – utilizing pancreatoscopy and EUS-PD – for complex postoperative leaks.

Endoscopy_UCTN_Code_TTT_1AR_2AG
